# Rubella outbreak in a Rural Kenyan District, 2014: documenting the need for routine rubella immunization in Kenya

**DOI:** 10.1186/s12879-015-0989-6

**Published:** 2015-06-27

**Authors:** Ian Njeru, Dickens Onyango, Yusuf Ajack, Elizabeth Kiptoo

**Affiliations:** Disease Surveillance and Response Unit, Ministry of Health, Nairobi, Kenya; Department of Health, Nakuru County, Nakuru, Kenya

**Keywords:** Rubella, Outbreak, Congenital Rubella Syndrome

## Abstract

**Background:**

Rubella infection has been identified as a leading cause of birth defects commonly known as Congenital Rubella Syndrome (CRS). Kenya does not currently have a rubella immunization program nor a CRS surveillance system. In 2014, a rubella outbreak was reported in a rural district in Kenya. We investigated the outbreak to determine its magnitude and describe the outbreak in time, place and person. We also analyzed the laboratory-confirmed rubella cases from 2010 to 2014 to understand the burden of the disease in the country.

**Methods:**

The Rubella outbreak was detected using the case-based measles surveillance system. A suspected case was a person with generalized rash and fever while a confirmed case was a person who tested positive for rubella IgM. All laboratory-confirmed and epidemiologically linked cases were line listed. The measles case-based surveillance database was used to identify rubella cases from 2010 to 2014.

**Results:**

A total of 125 rubella cases were line listed. Fifty four percent of cases were female. Case age ranged from 3 months to 32 years with a median of 4 years. Fifty-one percent were aged less than 5 years, while 82 % were aged less than 10 years. Six percent of the cases were women of reproductive age. All cases were treated as outpatients and there were no deaths. The number of confirmed rubella cases was 473 in 2010, 604 in 2011, 300 in 2012, 336 in 2013 and 646 in 2014.

**Conclusions:**

Analysis of Kenya rubella data shows that rubella is endemic throughout the country, and many outbreaks may be underestimated or undocumented. Six percent of all the cases in this outbreak were women of reproductive age indicating that the threat of CRS is real. The country should consider initiating a CRS surveillance system to quantify the burden with the goal of introducing rubella vaccine in the future.

## Background

The prevention of birth defects has been identified as global health priority [1]. Birth defects or congenital anomalies are a significant cause of morbidity, disability and mortality in many countries [2–5]. Rubella infection has been identified as one of the leading causes of birth defects globally [1].

Rubella, sometimes called German measles or three-day measles, is a contagious viral disease. The infection is usually mild with fever and rash. However, maternal infection during the first trimester of pregnancy can cause serious congenital malformations in the fetus, a condition which is known as Congenital Rubella Syndrome (CRS) [6].

The annual incidence of CRS in developing countries is estimated to be 110,000 cases per year, with a range of 14,000–308,000 [7]. The most common manifestation of CRS is deafness. Eye defects including glaucoma and retinopathy are common, and heart defects can also occur [8, 9].

The rubella vaccine contains a live attenuated (weakened) virus; immunization with two doses is highly effective to prevent rubella [10, 11]. The ultimate goal of rubella vaccination is to prevent the occurrence of CRS [12]. The World Health Organization (WHO) currently advocates for the use of rubella-containing vaccines (RCVs). RCVs are administered in monovalent form (rubella only) or in combinations such as Measles-Rubella (MR) or Measles-Mumps-Rubella (MMR) [13]. By December 2009, 130 WHO member states including two of 46 WHO Africa region member states used RCVs within their routine immunization systems [14].

Rubella vaccine is widely available through routine immunization programs in developed countries. However is it is not available in many developing countries including Kenya [15]. National rubella immunization programs in the developed countries utilize one of the following strategies: selective immunization of women, vaccination of infants, or a combined strategy [16]. When an infant vaccination strategy is adopted, there is need for sustained high coverage so as to ensure the susceptibility of adult women is not increased [17]. Although any of the three strategies could be used, it is important to include the vaccination of women of childbearing age [18, 19]. The vaccine is delivered through routine immunization or Supplemental Immunization Activities.

Rubella surveillance in Kenya is integrated within the national case-based measles surveillance system. Through this system, approximately 400 rubella cases are confirmed annually [20]. There is poor understanding of the burden of rubella infection and its prevalence among pregnant women in Kenya. A few studies have sought to establish the burden of rubella in Kenya. These studies, two in pregnant women and one in children were conducted in diverse parts of the country [20–22]. The study in pre-primary and primary school children identified an overall rubella sero-positivity of 80 %, with the highest sero-positivity (94 %) detected among children aged 14–20 years [20]. The studies in pregnant women showed that approximately 7 % were susceptible to rubella infection [21, 22].

Although rubella cases are detected every year through the measles surveillance system, a rubella outbreak in Kenya has not previously been described in the published literature. In 2014, a rubella outbreak was reported in Njoro, a rural District in Rift Valley Region of Kenya. We investigated the outbreak to determine its magnitude and describe the outbreak in time, place and person. We also analyzed laboratory confirmed rubella cases in the last 5 years (2010–2014) in order to fully define the burden of the disease in the country.

## Methods

### Study site

The study was conducted in Njoro district, located in Nakuru County, 150 km West of Nairobi (the capital city of Kenya). Njoro district is one of the nine districts in Nakuru County and has a population of 184,859.

### Study design

We did a retrospective analysis of data collected during the outbreak which occurred from March to May 2014.

### Laboratory investigations

Cases were identified through the measles case-based surveillance system which is also a passive surveillance system for rubella. Attending clinicians identified cases of fever and macula-papular rash during triage. Those who met the suspect case definition were notified to the district surveillance officer who completed a case-based surveillance form. The case-based surveillance form was sent to the national level of the Ministry of Health, while a copy was submitted to the laboratory with the blood specimen. Blood specimens were collected from the first six suspected cases. All six specimens were initially tested for measles-specific immunoglobulin M (IgM) antibody using a standard enzyme-linked immunosorbent assay (ELISA). All six specimens were negative for measles IgM, thus, they were tested for rubella-specific IgM antibody using ELISA. The laboratory testing was performed at Kenya Medical Research Institute (KEMRI) laboratory which is accredited by the WHO.

### Case definition

A suspected case of rubella was defined as anyone who presented with fever and maculo-papular rash from Njoro District from 1^st^ March 2014 to 15^th^ May 2014. A confirmed case was one that tested positive for rubella IgM. A probable case was one that had close contact with another case. As was the routine with measles and rubella surveillance in the country, once 5 or more samples from one district were laboratory-confirmed, it was no longer considered necessary to test all the samples. Therefore, all subsequent cases that presented with fever and maculo-papular rash were line-listed as rubella cases, provided they were epidemiologically linked to another case. An epidemiological link was defined as having been in close contact with someone with similar symptoms within the last 3 weeks. Close contact was defined as someone who lived in the same household, attended the same school or played together with the case.

### Data collection and analysis

Rubella cases were entered into an Excel line list during the outbreak. The line list captured socio-demographic variables including name, age, sex, date of onset, date seen at the facility, residence, laboratory results and outcome of illness. Information on measles and rubella vaccination status for each case was also collected. Data analysis was done using Epi Info version 7.

The study was approved by the Ministry of Health as part of routine surveillance and outbreak investigations and was therefore exempted from ethical committee review.

## Results

**Fig. 1:**
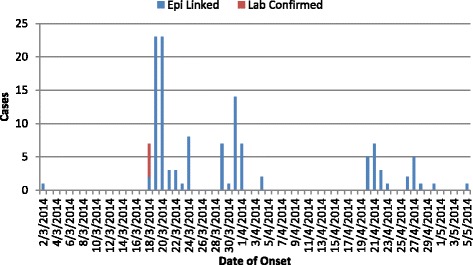
Epicurve of Rubella outbreak, Njoro District, Kenya, 2014

All 6 samples taken for laboratory confirmation on the first day of the outbreak (18th March 2014) tested positive for rubella IgM. Subsequently, a total of 125 rubella cases were line listed. The index case for this outbreak was believed to be a 2 year old child who accompanied his parents to another district that had reported laboratory- confirmed rubella cases, and returned to his home district on 1st March 2014. This child developed fever and rash on 2nd March 2014. Although the index case was not laboratory-confirmed, two cases developed rashes after contact with him, and were subsequently laboratory-confirmed. The peak of the outbreak occurred on 20th March 2014 while 38 % of all cases occurred in the first 3 days of the outbreak. This was followed by a gradual decline but with several intermittent smaller peaks. The last case occurred on 5th May 2014 (Fig. 1).

**Fig. 2:**
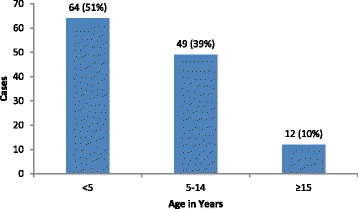
Age distribution of Rubella outbreak cases, Njoro District, Kenya, 2014

Fifty four percent of all cases were female with the age ranging from 3 months to 32 years with a median of 4 years. Fifty one percent of the cases were aged less than 5 years while 82 % were aged less than 10 years. A total of 7 cases (6 % of all cases) occurred in women of reproductive age (15 to 49 years) (Fig. 2). The outbreak started in one village but quickly spread to neighboring villages. In total, 15 villages reported cases; the number of reported cases ranged from 2 to 22 cases per village. Seventy five percent of children less than 5 years reported having been vaccinated against measles while none had been vaccinated against rubella. All cases were treated as outpatients and there were no deaths.

Using the passive rubella surveillance system where all measles negative samples are tested for rubella IgM, the number of rubella positive cases in Kenya varies by year, month and geographical location. The number of confirmed rubella cases was 473 in 2010, 604 in 2011, 300 in 2012, 336 in 2013 and 646 in 2014. Thirty two percent of the cases were aged less than 5 years while 80 % were aged less than 10 years. Fifty five per cent of the cases were female with only 3 % of them being in the reproductive age group. Overall, only 1.6 % of the cases were women of reproductive age group.

**Fig. 3:**
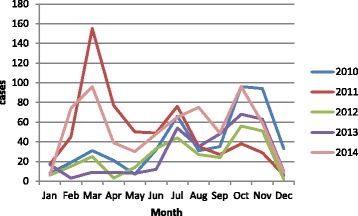
Distribution of laboratory confirmed Rubella cases by month and year, Kenya, 2010–2014

**Fig. 4:**
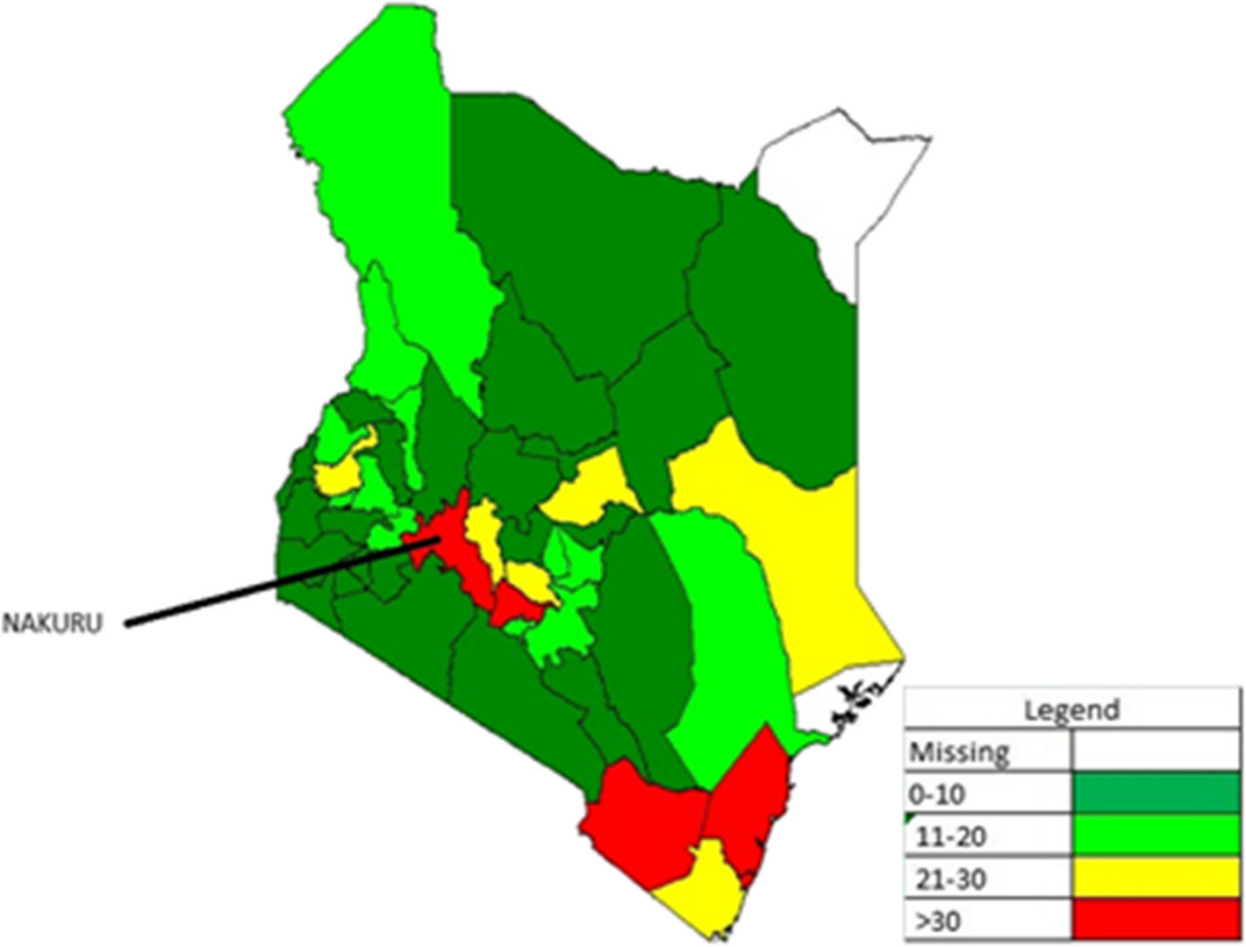
Distribution of laboratory confirmed Rubella cases, Kenya, 2014

The cases varied by month in all the years but common peaks were noted in the months of March, July and October (Fig. 3). Cases were reported from a majority of the 47 counties, with 40 counties (85 %) reporting cases in 2010, 46 counties (96 %) in 2011, 40 counties (85 %) in 2012, 39 counties (83 %) in 2013 and 45 counties (96 %) in 2014. The highest number of cases (646) was reported in 2014 and included 45 (96 %) counties. The number of cases varied by county with Nakuru County reporting one of the highest cases in 2014 (Fig. 4).

## Discussion

This is the first rubella outbreak detected, investigated and documented in the country. Previous rubella cases captured by measles case-based surveillance system have been sporadic throughout Kenya. The index case in this outbreak was a child who visited another district experiencing an outbreak. We attributed this outbreak to the fact that our country does not currently provide RCV in the National Immunization Program; therefore most of the children were susceptible to the disease.

Studies in other countries not providing RCVs have also demonstrated widespread transmission [23–25]. An example of such an outbreak occurred in India when the country was not providing RCV in their National Immunization Program [26]. Where outbreaks have occurred in countries which immunize against rubella, cases have been restricted to pockets of non-immunized individuals [27–33].

Many cases of rubella are identified every year in Kenya by the measles surveillance system (Figs. 3 and 4). However, there is currently no effective intervention for rubella in the country; this is in stark contrast to measles outbreaks which are promptly followed up with vaccination campaigns.

With regard to seasonality, this outbreak started in March which is one of the 3 months with the highest number of rubella cases every year. The other months with large numbers of rubella cases are July and October (Fig. 3). These months are characterized by a rainy season and cold weather, resulting in increased cases of respiratory disease; this could explain the high number of rubella cases reported during these months.

The data we obtained demonstrates that over the years the country has consistently detected numerous rubella cases, in spite of the low sensitivity of the passive surveillance system. The high proportion of Kenya counties reporting rubella cases is an indicator of wider transmission. Additionally, our data confirms the assertion of Kombich *et al.* that the virus is widely prevalent among children in Kenya [20].

Six percent of the outbreak cases and 1.6 % of the routine rubella data were women of reproductive age. The low percentage of affected women as indicated by the routine data could be an underestimation of the true burden as this is a passive surveillance system and many adult women are unlikely to seek health care when the disease is mild or asymptomatic.

Kenya like many other African countries does not currently provide RCV within the national immunization program, and a surveillance system for CRS does not currently exist. In order to document the burden of CRS, the country should consider establishing a CRS surveillance system. The country could also consider adopting the WHO policy on rubella vaccination which currently recommends combining measles and rubella control strategies and planning efforts which focus on the widespread use of measles-rubella vaccine [34].

This outbreak report has several limitations. First, the passive rubella surveillance system may have grossly underestimated the magnitude of the outbreak. Many asymptomatic rubella cases or those with mild symptoms may have been missed as they may not have sought healthcare. Secondly, the annual number of nationally confirmed rubella cases may also be a gross underestimation of the total burden of cases, as these data are derived from the measles surveillance system and not a rubella-specific surveillance system. Thirdly, many adults including women of reproductive age could also have been missed by the surveillance system as many may not have sought health care owing to the mild nature of the disease.

## Conclusions

This outbreak documented that large rubella outbreaks occur in countries without rubella vaccination programs. Evaluation of 5 years of rubella data shows that the disease is endemic throughout the country, therefore outbreaks may be largely underestimated and undocumented. The fact that 6 % of all cases in this outbreak were women of reproductive age indicates that the threat of CRS is real. It is therefore important for Kenya to initiate CRS surveillance to quantify the actual burden with the goal of introducing rubella vaccine in the future.
